# Population-level variation in gut bifidobacterial composition and association with geography, age, ethnicity, and staple food

**DOI:** 10.1038/s41522-023-00467-4

**Published:** 2023-12-12

**Authors:** Jing Lu, Li Zhang, Hao Zhang, Yutao Chen, Jianxin Zhao, Wei Chen, Wenwei Lu, Mingkun Li

**Affiliations:** 1 State Key Laboratory of Food Science and Technology, Jiangnan University, Wuxi, 214122 China; 2 School of Food Science and Technology, Jiangnan University, Wuxi, 214122 China; 3 Key Laboratory of Genomic and Precision Medicine, Beijing Institute of Genomics, Chinese Academy of Sciences, and China National Center for Bioinformation, Beijing, 101300 China; 4 National Engineering Research Center for Functional Food, Jiangnan University, Wuxi, 214122 China; 5 International Joint Research Laboratory for Pharmabiotics & Antibiotic Resistance, Jiangnan University, Wuxi, 214122 China; 6 University of Chinese Academy of Sciences, Beijing, 100049 China

**Keywords:** Microbiome, Microbial ecology

## Abstract

Bifidobacteria are key gut commensals that confer various health benefits and are commonly used as probiotics. However, little is known about the population-level variation in gut bifidobacterial composition and its affecting factors. Therefore, we analyzed *Bifidobacterium* species with amplicon sequencing of the *groEL* gene on fecal samples of 1674 healthy individuals, who belonged to eight ethnic groups and resided in 60 counties/cities of 28 provinces across China. We found that the composition of the bifidobacterial community was associated with geographical factors, demographic characteristics, staple food type, and urbanization. First, geography, which reflects a mixed effect of other variables, explained the largest variation in the bifidobacterial profile. Second, middle adolescence (age 14–17) and age 30 were two key change points in the bifidobacterial community development, and a bifidobacterial community resembling that of adults occurred in middle adolescence, which is much later than the maturation of the whole gut microbial community at approximately age 3. Third, each ethnicity showed a distinct bifidobacterial profile, and the remarkable amount of unknown *Bifidobacterium* species in the Tibetan gut suggested undiscovered biodiversity. Fourth, wheat as the main staple food promoted the flourish of *B. adolescentis* and *B. longum*. Fifth, alpha diversity of the bifidobacterial community decreased with urbanization. Collectively, our findings provide insight into the environmental and host factors that shape the human gut bifidobacterial community, which is fundamental for precision probiotics.

## Introduction

Bifidobacteria, a group of Gram-positive, heterofermentative, non-motile, non-spore-forming microorganisms, are among the first microbial colonizers of the human gut. They are considered key commensals with health-promoting functions, contributing to the homeostasis of the gut microenvironment, development of the immune system, and utilization of dietary components in both infants and adults^1,2^. Moreover, bifidobacteria are well-known probiotics, with some strains being used as bioactive components in functional foods, food supplements, and drugs^3,4^.

The abundance and diversity of bifidobacteria vary throughout human life and are associated with multiple factors, with age being the most significant. Bifidobacteria dominate the infant gut, and their abundance can be influenced by the mode of delivery, type of feeding, and antibiotic exposure, among other factors^5^. They can comprise more than 60% of the total bacterial community in infants^6^, with *Bifidobacterium breve, B. bifidum, B. longum spp. longum, B. longum spp. infants and B. pseudocatenulatum* being the dominants^7–9^. However, in adults, bifidobacteria decreases to less than 10% of the total bacterial community^10–12^, and they are mainly *B. adolescentis, B. longum, B. pseudocatenulatum, and B. catenulatum*^13–15^. In the elderly, bifidobacterial abundance and diversity further decrease. Although these general patterns of bifidobacterial composition have been revealed in different age groups, the continuous development process along the lifespan remains unclear.

Diet plays a critical role in modulating the bifidobacterial community in the human gut, with non-digestible carbohydrates (known as prebiotics) being selectively fermented by bifidobacteria and other beneficial microorganisms. The proportion of genes involved in carbohydrate metabolism in bifidobacteria is ~30% higher than that in most gut microbes, and the ability to metabolize different carbohydrates is species- and strain-dependent^3,16^, suggesting a vigorous and diversified response to the diet of *Bifidobacterium* spp. For example, the flourishing of bifidobacteria in adults has been repeatedly linked to high wheat intake and high FODMAP (fermentable oligosaccharides, disaccharides, monosaccharides, and polyols) diet^10,17–19^. However, the alteration of bifidobacterial composition in response to various diets and related health outcomes is still largely unknown. Furthermore, differences in the gut bifidobacterial profile likely exist between large-scale geographical regions. For example, *B. longum, B. adolescentis, and B. bifidum* were the major species in the Italian centenarians^20^, whereas *B. longum* and *B. dentium* were the dominants in the Chinese centenarians^21^. However, the geographic scale at which variation in the bifidobacterial community occur, and the extent to which sub-factors of geography, such as climate, topography, lifestyle, and long-term diets, contribute to the variation remain unknown.

As the most widely used probiotics, the persistence and consequent health benefits of orally administered bifidobacteria largely depend on the niche opportunity in the gut. A previous study showed that *B. longum* AH1206 was able to colonize when the pre-treatment gut had a low abundance of resident *B. longum* and specific carbohydrate utilization genes^22^. This highlights the importance of clear bifidobacterial and microbial background (i.e., species and functions already exist in the gut) in the precise design and application of probiotic bifidobacteria, which requires a comprehensive understanding of bifidobacterial composition of different populations and its affecting factors. However, large cohort studies investigating population-level configurations of gut bifidobacteria are still missing.

In this study, we aimed to characterize the population-level variation in the gut bifidobacterial community and identify factors associated with it. We used an amplicon sequencing method targeting the *groEL* gene, which determines bifidobacteria at the species level^23^, and illuminated diverse associations between the bifidobacterial composition and geographical factors, demographic factors, staple food, as well as urbanization. Additionally, by conducting a comparative genome analysis of bifidobacterial isolates, we were able to provide causal insights into some of the associations.

## Results

### Overview of samples and data

**Fig. 1:**
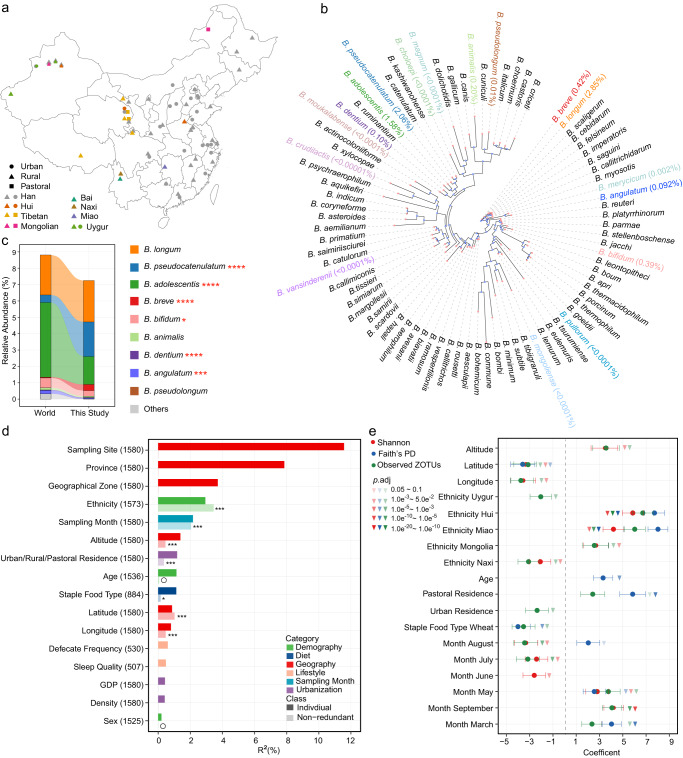
Diversity of *Bifidobacterium* species in the Chinese gut and associated covariates. **a** Geographical distribution of sampling sites, ethnic groups, and urbanization status. **b** The phylogenetic tree of *groEL* gene of *Bifidobacterium* species. A total of 82 *Bifidobacterium* species with genome deposited in NCBI database were included, and one sequence from each species was randomly selected from the NCBI database accessed on 20 August 2021. The 17 species found in this study are highlighted by different colors, and their mean relative abundances are shown in brackets. **c** Composition of the bifidobacterial community in this study and the World data. *p.adj < 0.1, ***p.adj < 0.01, ****p.adj < 0.001, linear regression with adjustment for age and sex. This study, *n* = 1413; World, *n* = 4516. **d** Covariates correlated with the beta diversity of the gut bifidobacterial community estimated with Bray–Curtis dissimilarity. Nineteen variables having more than 500 samples with non-missing data were analyzed using dbRDA separately, and the variance explained by each of these variables is indicated with darker colors; 16 covariates with *p*.adj < 0.1 are shown. Nine representative variables having >800 samples with non-missing data were analyzed using a stepwise dbRDA, and the non-redundant variance explained by each of these variables is indicated with lighter colors; **p.adj < 0.05, ***p.adj < 0.01, and nonsignificant covariates are indicated with an open circle; *n* = 884. The number of samples is indicated in brackets following the name of each covariate. **e** Covariates associated with the alpha diversity of the gut bifidobacterial community. The nine representative variables used in **d** were subjected to *z* score normalization and then analyzed using a ridge regression model. Error bars indicate standard errors of the coefficients. *n* = 884.

The study included 1674 volunteers without apparent diseases (referred to as “healthy”, 712 males and 813 females, age range 0.01–103 years with a median of 41), which comprised 1349 Han Chinese, 309 individuals from seven ethnic minority groups (Tibetan, 93; Hui, 72; Miao, 36; Naxi, 33; Uygur, 31; Mongolian, 30; Bai, 14) and 16 individuals without ethnic information (Supplementary Table 1). Participants resided in 60 counties/cities across 28 provinces in China (Fig. 1a). The cohort was a subset of a previously reported cohort^10^, whose gut microbiota have been profiled with 16 S rRNA gene sequencing. Fecal samples and participant metadata, including geography, demography, anthropometrics, diet, lifestyle, urbanization status, and other factors (20 variables in total, Supplementary Table 1), were collected as previously described^10^.

Bifidobacterial species profiling was performed by sequencing the *groEL* gene. A total of 603 zero-radius operational taxonomic units (ZOTUs), accounting for 74.30% of the total reads, were assigned to 17 bifidobacterial species, and 824 ZOTUs, accounting for 11.05% of the total reads, were assigned to *Bifidobacterium* without species-level resolution (Supplementary Table 2). A median of 17,249 reads belonging to *Bifidobacterium* was obtained per sample (range 2,000–144,728). The relative abundance of each *Bifidobacterium* species was calculated by normalizing its reads to the total bifidobacterial reads and multiplying by the relative abundance of *Bifidobacterium* in the total bacterial community, which was inferred from 16 S rRNA gene sequencing.

### Diversity of gut *Bifidobacterium* species and associated covariates

The 17 species found in our study were phylogenetically diverse among 82 *Bifidobacterium* species that had already been recognized in nature, reflected by dispersed distribution on the phylogenetic tree built on the *groEL* gene (Fig. 1b, Supplementary Fig. 1a). Nine of the 17 species were abundant (mean relative abundance >0.01%), including *B. longum* (2.85%), *B. pseudocatenulatum* (2.06%), *B. adolescentis* (1.58%), *B. breve* (0.42%), *B. bifidum* (0.39%), *B. animalis* (0.20%), *B. dentium* (0.10%), *B. angulatum* (0.092%), and *B. pseudolongum* (0.01%). We next compared the bifidobacterial composition in this study to that in the existing metagenomic data worldwide (referred to as “World”), which was represented by 4,516 healthy individuals from 14 countries (Supplementary Table 3, and the comparable age distribution of the two datasets was shown in Supplementary Fig. 2a). There were many overlaps in abundant species between the two datasets, except that two worldwide abundant species, *B. catenulatum* (0.31%) and *B. ruminantium* (0.01%) were not detected in our data. Besides, we observed higher levels of *B. pseudocatenulatum*, *B. breve*, *B. dentium*, and *B. pseudolongum*, whereas lower levels of *B. adolescentis*, *B. bifidum*, and *B. angulatum* were observed in our cohort compared to the World data (*p*.adj <0.1, linear regression, Fig. 1c).

To identify covariates associated with beta diversity (inter-individual dissimilarities) of the bifidobacterial community, we calculated Bray–Curtis dissimilarity and Jensen–Shannon divergence (JSD), and applied distance-based redundancy analyses (dbRDA) for 19 covariates separately. Sixteen covariates significantly explained the variation in bifidobacterial community when assuming independence (*p*.adj < 0.1), with a group of geographical factors (sampling site, province, and geographical zone) explaining the largest variation (Fig. 1d, Supplementary Fig. 2b). The bifidobacterial composition also changed gradually in proximal locations, as demonstrated by the significant correlation between the distance matrices and geographic distance (*p* = 0.01 for Bray–Curtis dissimilarity and *p* = 0.001 for JSD, Mantel test). Subsequently, we selected representative variables of different metadata categories and incorporated them to a stepwise dbRDA model. A demographical factor, ethnicity, turned out to be the most explanatory variable, followed by sampling month, geographical factors (latitude, longitude, and altitude), urbanization status (urban/rural/pastoral residence), and staple food type belonging to the diet category (Fig. 1d, Supplementary Fig. 2b). In contrast, the impact of sex and age were not significant, although their significance was identified in the univariate model. For age, it might be due to different age distributions in the two models, since the stepwise dbRDA model only included subjects having information on the staple food type, and most children were thus excluded.

Regarding alpha diversity (intrinsic microbial diversity), we incorporated three indices, Observed ZOTUs, Faith’s Phylogenetic Diversity (Faith’s PD), and Shannon index, with the above-mentioned nine representative variables to ridge regression models, respectively. Among the geographical factors, latitude was negatively correlated with all three indices, indicating an increase in bifidobacterial richness, evenness, and phylogenetic diversity with lower latitudes. Besides, altitude was positively correlated with Observed ZOTUs and Shannon index, whereas longitude showed the opposite pattern. Among the demographical factors, age is positively associated with Faith’s PD, and most ethnic minority groups showed significantly higher or lower alpha diversity compared to Han Chinese, however, sex was not associated with either of the three indices. For urbanization, pastoral residence was positively associated with all three indices, whereas urban residence was negatively associated with Observed ZOTUs (compared to rural). In addition, wheat as the main staple food was associated with lower Observed ZOTUs and Faith’s PD (compared to rice as the main staple food); different sampling months also had influences on alpha diversity (Fig. 1e). Further characterization of the associations between the bifidobacterial community and important covariates including geography, age, ethnicity, staple food type, and urbanization was conducted in the following sections.

### Geographical distribution of gut *Bifidobacterium* species

**Fig. 2:**
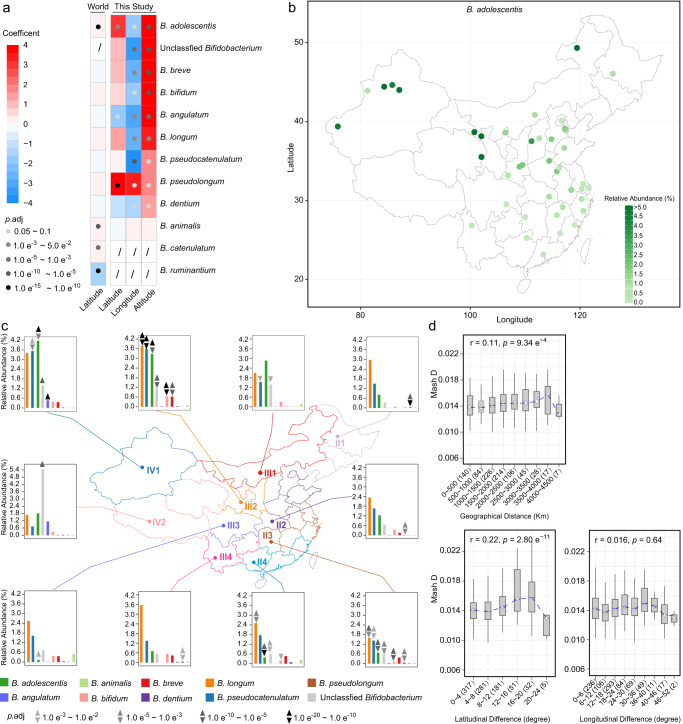
Geographical distribution of gut *Bifidobacterium* species. **a** The association between geographical factors and the abundance of *Bifidobacterium* species. Coefficient and *p*.adj values from ridge regression for this study and those from multiple linear regression for the World data are shown. *z* score normalization of continuous variables and relative abundances was performed prior to regression analyses. This study, *n* = 884; World, *n* = 4516. **b** Relative abundance of *B. adolescentis* in different sampling sites across China. Each dot represents a sampling site; the color of the dot indicates the mean value in each site; sampling sites with at least 15 samples are shown. *n* = 1528. **c** Relative abundances of the nine most abundant *Bifidobacterium* species as well as unclassified *Bifidobacterium* in different geographical zones in China. The 10 geographical zones are indicated by different colors, and the mean relative abundances of *Bifidobacterium* species in each zone are shown in the bar plot. Geographical zone-specific species (*p*.adj < 0.01) are indicated by triangles (linear regression, *n* = 1413) and inverted triangles (ridge regression, *n* = 884) with different greyscale. **a**, **c** Age and sex were included as confounding factors in linear regression models; age, sex, ethnicity, sampling month, staple food type, and urban/rural/pastoral residence were included in ridge regression models in a; age, sex, ethnicity, sampling month, and urban/rural/pastoral residence were included in ridge regression models in **c**. **d** Mash distance between *B. pseudocatenulatum* genomes in different geographical groups. The center line of the boxplot represents the median, box limits represent upper and lower quartiles, and whiskers represent 1.5× interquartile range. The number of site pairs in each distance range is indicated in brackets. The associations between geographical distance and Mash distance were evaluated using Pearson’s correlation tests, and the coefficient and *p* values are shown.

To investigate the geographical distribution patterns of gut *Bifidobacterium* species, we correlated the relative abundance of each species with latitude (23 °N to 50 °N), longitude (75 °E to 130 °E), and altitude (3–3853 km) using a ridge regression model. All nine abundant species except *B. animalis*, as well as unclassified *Bifidobacterium*, were positively correlated with altitude (*p*.adj < 0.1); of them, *B. pseudolongum* was positively correlated with longitude (i.e., the relative abundance increased from west to east) whereas the others were negatively correlated with longitude. In terms of latitude, *B. adolescentis* and *B. pseudolongum* showed a positive correlation with it (i.e., the relative abundance increased from north to south), whereas *B. angulatum* showed the opposite (Fig. 2a, b). Notably, the positive correlation between the abundance of *B. adolescentis* and latitude was also observed in the World data (from 62 °N to 20 °S, Fig. 2a).

To intuitively illustrate the geographical characteristics of bifidobacterial composition, we visualized the distribution of the nine abundant species across ten geographical zones in China, which differ in climate, topography, etc. (Fig. 2c). Remarkably, IV2, where the Tibetan Plateau is located, showed a higher proportion of unclassified *Bifidobacterium* (mean relative abundance 5.5%, compared to 0.3%–1.4% in other geographical zones), indicating the presence of a significant amount of undiscovered biodiversity in that specific area. In addition, IV1 and IV2 showed higher *B. angulatum*, with relative abundances of 0.45% and 1.15%, respectively, whereas it was lower than 0.04% in other geographical zones. To identify geographical zone-specific features, we further applied two regression models, linear regression with adjustment for age and sex, as well as ridge regression with adjustment for multiple confounding factors (age, sex, ethnicity, rural/urban/pastoral residence, sampling month). The two models revealed 24 and 23 geographical zone-specific features, respectively (of which 20 were identified by both models, *p*.adj <0.01, Fig. 2c). Interestingly, the above-mentioned significance for unclassified *Bifidobacterium* and *B. angulatum* was not detected when controlling for multiple confounding factors, suggesting that part of the effect of geography on bifidobacterial composition was confounded by other factors.

Given the geographical variation in the abundance of various species, we further investigated if the genomes of the *Bifidobacterium* species also differed by geographical locations. We obtained 26 isolates of *B. adolescentis* from 26 fecal samples from 11 sampling sites, as well as 45 isolates of *B. pseudocatenulatum* from 44 fecal samples from 22 sampling sites (Supplementary Table 4). We found that the Mash distances between genomes of *B. pseudocatenulatum* isolates significantly increased with geographical distances (*p* < 0.001, Pearson correlation, Fig. 2d), but this tendency was not evident for *B. adolescentis* (*p* = 0.55, Supplementary Fig. 3). We further dissected the geographical distance to latitudinal and longitudinal differences, and found that the latitudinal difference positively correlated with the genomic distance between *B. pseudocatenulatum* isolates (*p* < 10^−10^), but not the longitudinal difference (*p* = 0.64, Fig. 2d).

### Development of the bifidobacterial community with aging

**Fig. 3:**
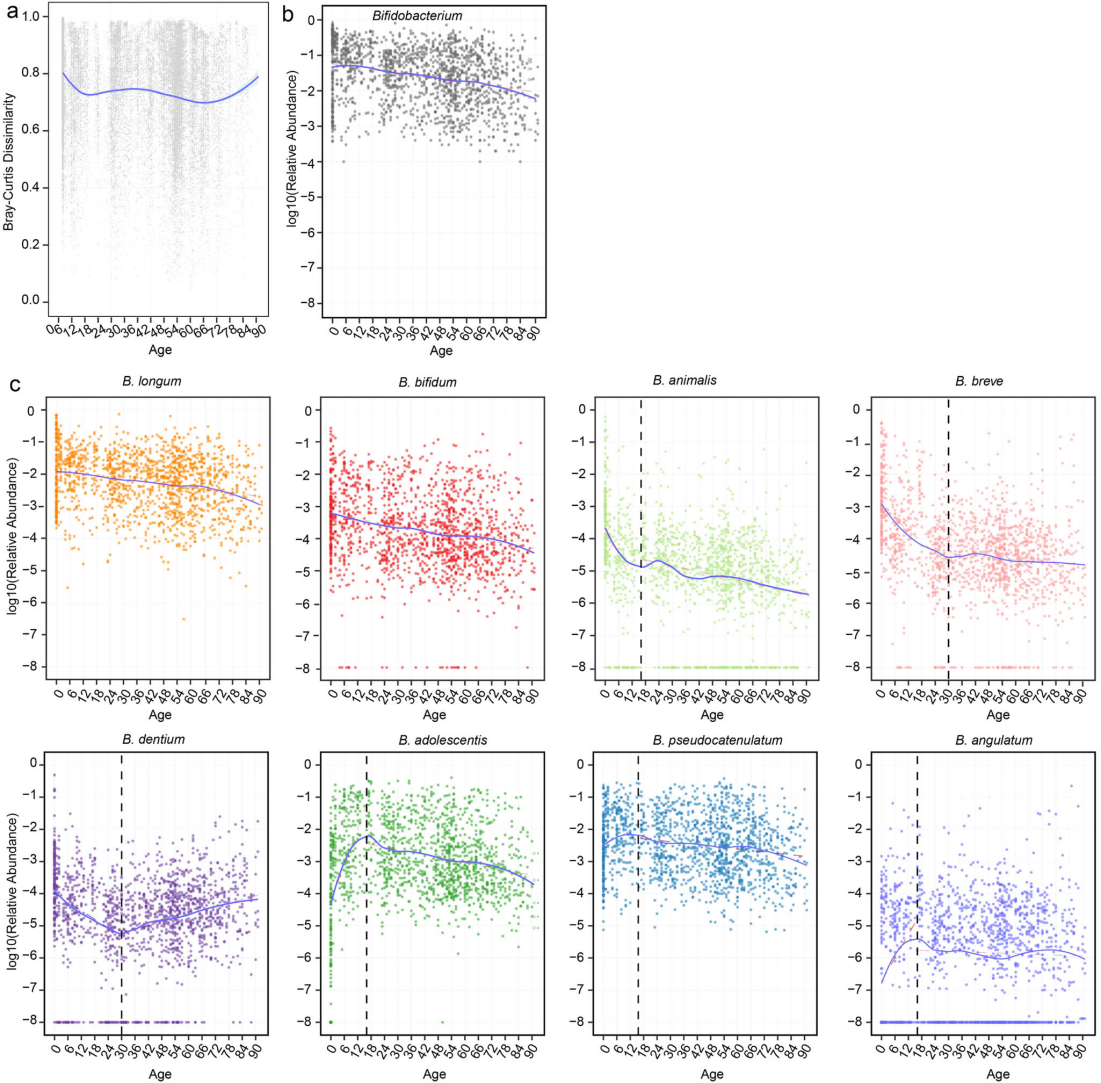
Changes of the gut bifidobacterial community along aging. **a** Bray–Cutis dissimilarity between bifidobacterial communities of adjacent ages. Each dot represents the distance between one bifidobacterial community at a specific age and another bifidobacterial community at the age one year younger. **b** Relative abundance of *Bifidobacterium*. **c** Relative abundance of each *Bifidobacterium* specie. **b**, **c** each dot represents a sample. Fitting curves of loess regression models are indicated with blue lines, and those of linear regression models are indicated with red dash lines. Black vertical dash lines indicate key change points (age 16 or 30) in the development of bifidobacterial community. *n* = 1536.

To investigate the development of the bifidobacterial community throughout the lifespan, we calculated the Bray-Curtis dissimilarity between bifidobacterial communities of adjacent ages. Our results revealed that the Bray–Curtis dissimilarity decreased sharply from birth to middle adolescence (age 14–17, Fig. 3a), suggesting that the bifidobacterial community reaches maturity during middle adolescence. The Bray–Curtis dissimilarity underwent a gentle fluctuation during the early and middle adulthood, and started to increase rapidly after age 70. In contrast, the abundance of total *Bifidobacterium* decreased linearly with age (Fig. 3b).

The nine abundant *Bifidobacterium* species showed markedly different aging patterns (Fig. 3c). *B. longum* and *B. bifidum* displayed a linear decline with age. The abundance of *B. animalis* exhibited a rapid drop before middle adolescence and a much slower drop thereafter. The abundance of both *B. breve* and *B. dentium* dropped rapidly from birth to age 30, but their changing trends differed afterwards, with the former remaining stable whereas the latter increasing. *B. adolescentis* increased sharply from birth to middle adolescence, followed by a rapid decline. *B. pseudocatenulatum* and *B. angulatum* also demonstrated a rising tendency before middle adolescence, but only minor changes thereafter (Fig. 3c). *B. pseudolongum* did not show any clear changing trend (Supplementary Fig. 4a). Taken together, our results suggest that middle adolescence (age 14–17) and age 30 are key change points in the development of the bifidobacterial community. Similar patterns were observed in the World data for *B. bifidum*, *B. adolescentis*, and *B. dentium* (Supplementary Fig. 4b).

### Ethnicity-specific characteristics of the bifidobacterial community

**Fig. 4:**
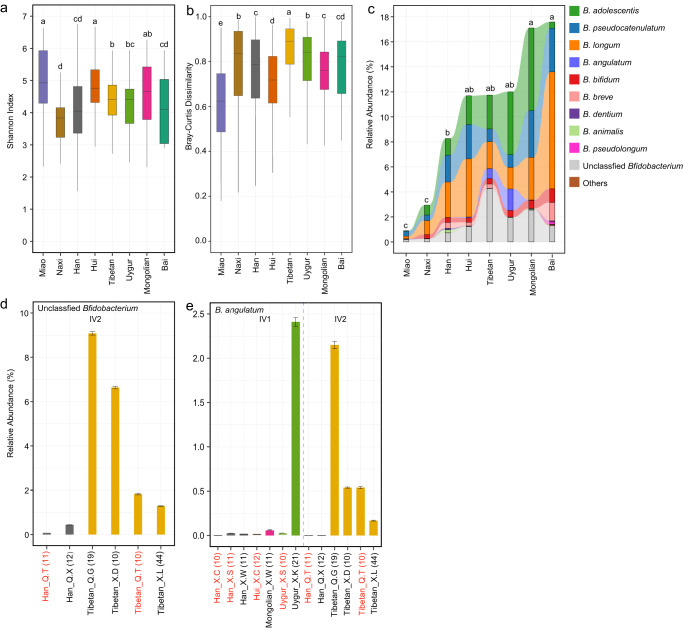
Ethnicity-specific characteristics of the gut bifidobacterial community. **a** Alpha diversity represented by Shannon Index. **b** Bray–Cutis dissimilarity within each ethnic group. **a**, **b** The center line of the boxplot represents the median, box limits represent upper and lower quartiles, and whiskers represent 1.5× interquartile range. **c** Composition of the bifidobacterial community in each ethnic group. Mean values are shown. **a**–**c** Different letters on the top indicate significant differences in alpha diversity, beta diversity, and relative abundance of total *Bifidobacterium* (*p*.adj < 0.1, one-way ANOVA and Fisher’s Least Significant Difference test). Han, *n* = 1349; Tibetan, *n* = 93; Bai, *n* = 14; Miao, *n* = 36; Naxi, *n* = 33; Uygur, *n* = 31; Hui, *n* = 72; Mongolian, *n* = 30. **d** Relative abundances of unclassified *Bifidobacterium* in different ethnic groups per sampling site in geographical zone IV2. **e** Relative abundances of *B. angulatum* in different ethnic groups per sampling site in IV1 and IV2. **d**–**e** The height of columns indicates mean value, and error bars indicate standard deviations. Labels on the horizontal axis indicate the name of ethnic groups and sampling sites, as well as the number of samples included; sampling sites having two ethnic groups were indicated in red.

The total abundance, alpha diversity, beta diversity, and composition of the bifidobacterial community were found to substantially vary among the Han Chinese and seven ethnic minority groups (Fig. 4a–c, Supplementary Fig. 5). Among them, Miao showed the lowest bifidobacterial abundance and intra-group divergence, but the highest alpha diversity. In contrast, Naxi, whose total abundance was the second lowest, showed relatively high intra-group divergence and the lowest alpha diversity.

Tibetan and Uygur showed remarkably high levels of *B. angulatum*, and Tibetan also showed a fairly high level of unclassified *Bifidobacterium* (Fig. 4c). Considering that the two ethnic minority groups mainly resided in geographical zones IV1 and IV2 respectively, which also showed high levels of *B. angulatum* (and unclassified *Bifidobacterium* in IV2, Fig. 2c), we aimed to tease apart the effect of ethnicity and geography. We dissected the data from IV1 and IV2 by each sampling site and ethnicity group. The results showed that the high abundance of unclassified *Bifidobacterium* was associated with Tibetan but not the IV2 region, as the relative abundance of unclassified *Bifidobacterium* was high in Tibetan from all four sampling sites in IV2 (mean value 9.08%, 6.63%, 1.83%, and 1.28%, respectively), whereas it was less than 0.44% in Han Chinese from two sites in IV2 (Fig. 4d). Regarding *B. angulatum*, Tibetan from four sampling sites in IV2 had mean relative abundances of 2.15%, 0.54%, 0.54%, and 0.16%, respectively, and the first site had mainly Tibetan residing whereas the other three sites had multiple ethnic groups co-residing. Uygur from one site in IV1, where only this one ethnic group resided, had 2.42% *B. angulatum*; in contrast, it was only 0.01% in Uygur from another site, where Uygur and Han co-resided. Han Chinese from six sites, Hui from one site, and Mongolia from one site in IV1 and IV2 all had *B. angulatum* below 0.06% (Fig. 4e). These results suggest that the high abundance of *B. angulatum* is mainly driven by ethnicity Tibetan and Uygur, and co-residence with other ethnic groups or geography also contribute to the varied abundance of this species.

### Staple food type influenced the composition of the bifidobacterial community

**Fig. 5:**
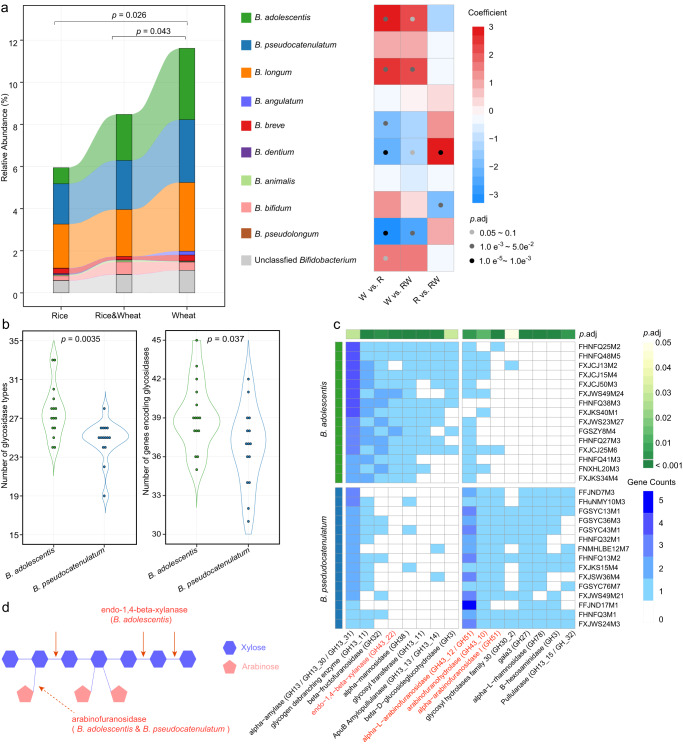
Effect of staple food type on the gut bifidobacterial community and its association with glycosidase-encoding genes. **a** Composition of the bifidobacterial community in each staple food group. Ridge regression (with adjustment for age, sex, ethnicity, altitude, latitude, longitude, sampling month, and urban/rural/pastoral residence) was used to assess differences in the relative abundance of total *Bifidobacterium* between groups (indicated with *p* values on the top of the bar plot), as well as differences in the relative abundance of each *Bifidobacterium* species between groups (indicated with coefficient and *p*.adj values in the heatmap). In the bar plot, mean values are displayed. R, rice, *n* = 311; W, wheat, *n* = 354; RW, rice & wheat, *n* = 219. **b** Number of glycosidase types (left) and number of genes encoding glycosidases (right) of *B. adolescentis* and *B. pseudocatenulatum* isolates. *p* values from Mann–Whitney tests are shown. **c** Differential glycosidase-encoding genes between *B. adolescentis* and *B. pseudocatenulatum* isolates. *p*.adj values from Mann–Whitney tests are shown. Glycosidases that hydrolyze arabinoxylan were highlighted in red. **d** Illustration of arabinoxylan degradation. Endo-1,4-beta-xylanase from *B. adolescentis* cleaves arabinoxylan by internally hydrolyzing the 1,4-beta-D-xylosidic linkage between xylose residues in the xylan backbone, and arabinofuranosidases from *B. adolescentis* and *B. pseudocatenulatum* remove arabinose substituents from the xylan backbone.

The participants were divided into three groups based on their long-term staple food preferences: wheat (white flour of common wheat), rice (white rice), and a combination of wheat and rice. Previous research has established that wheat intake promotes the expansion of *Bifidobacterium*^24–26^, which was also observed in this cohort (Fig. 5a). Analysis of the association between staple food type and bifidobacterial alpha and beta diversity (Fig. 1d, e) suggested that different species may respond differently. To investigate these effects, we performed ridge regression to compare each two groups, and found that the *Bifidobacterium* species could be grouped into three categories based on their responses to staple food: wheat-responders, including *B. adolescentis* and *B. longum*, which increased with wheat intake (*p*.adj values < 0.1 for at least two comparisons) and presented in high abundance in the gut; rice-responders, including *B. dentium* and *B. pseudolongum*, which increased with rice intake and presented in low abundance in the gut; non-responders, including *B. pseudocatenulatum*, *B. angulatum*, and *B. animalis* that showed no significance, as well as *B. bifidum* and *B. breve* that only showed moderate alterations in one comparison (Fig. 5a).

To further explore the correlation between gut bifidobacterial configuration and host staple food preference, we chose two abundant species, *B. adolescentis* as a strong wheat-responder and *B. pseudocatenulatum* as a non-responder, and randomly selected 15 isolates from each species for comparative genome analysis (Supplementary Table 4). We focused on 270 functional genes encoding glycosidases (EC 3.2.1), considering that dietary fibers from staple food are not digested by the host and left for microbial fermentation, and the amount and component of dietary fibers in wheat and rice differ significantly^27,28^. The *B. adolescentis* isolates had a higher number of glycosidases types and genes encoding glycosidases than the *B. pseudocatenulatum* isolates, indicating a higher capacity of carbohydrate catabolism. This is in line with previous literature^29^. We identified 16 glycosidase genes with differential gene counts between the two species (*p*.adj < 0.05, Mann–Whitney test, Fig. 5c). Notably, the genes responsible for arabinoxylan degradation differed between *B. adolescentis* and *B. pseudocatenulatum* isolates. First, 14 *B. adolescentis* isolates but none of the *B. pseudocatenulatum* isolates possessed genes encoding endo-1,4-beta-xylanase (EC 3.2.1.8), indicating that only *B. adolescentis* can cleave the xylan backbone; second, although *B. pseudocatenulatum* isolates showed a higher prevalence of genes encoding three arabinofuranosidases (EC 3.2.1.55), both species possessed this glycosidase and can thus remove arabinose substituents from the xylan backbone (Fig. 5c, d)^30^. These results imply that *B. adolescentis* has a higher efficiency of utilizing arabinoxylan, which constitutes 70% of the cell wall polysaccharide in white flour^31^, and this may account for the stronger response to wheat intake in *B. adolescentis* compared to *B. pseudocatenulatum*. Altogether, these findings suggest that the correlation between bifidobacterial composition and long-term staple food preferences may be partially explained by the varying capacity of *Bifidobacterium* species to hydrolyze specific dietary fibers.

### Association between urbanization and the gut bifidobacterial community

We compared the gut bifidobacterial community in 629 residents from 26 rural sites (area range 0.07–8.42 km^2^ with a median of 0.23 km^2^) in 16 provinces and 237 residents from 14 urban sites (area range 0.02–13.54 km^2^ with a median of 0.46 km^2^) in 13 provinces. Besides the differences in alpha diversity (Fig. 1e), urban residents showed slightly higher *B. animalis* and *B. pseudolongum* whereas lower *B. dentium* than rural residents (*p*.adj < 0.1, ridge regression, Supplementary Fig. 6), although the abundance of total *Bifidobacterium* did not differ between the two groups.

## Discussion

The gut microbiota plays an important role in human health, and bifidobacteria are among the most important commensal microbes and probiotics. While their genomics and functionality have been extensively studied^32–37^, the population-level variation in the gut bifidobacterial community has received limited attention. To address this gap, we conducted the largest cohort study to date investigating the configurations of gut bifidobacterial across a broad range of geography, climate, topography, age, dietary habits, and culture.

Our results revealed that geography was the primary factor influencing both the whole microbial community^10^ and the bifidobacterial community. Geography reflects a mixed effect of ethnicity, diet, lifestyle, urbanization etc., for example, altitude is linked to some ethnic minority groups living in the plateau, latitude is linked to different staple food, and longitude is linked to urbanization in this cohort. This may be the reason for its high degree of explanation of variation in the bifidobacterial community. After adjusting for multiple confounding factors, we still observed considerable effect of altitude, latitude, and longitude, indicating more underlying factors to be explored. Therefore, comprehensive metadata, especially detailed dietary information should be collected to disentangle the covariates in future studies.

Two demographic factors, ethnicity, and age, also significantly affected the bifidobacterial community. In our previous study, the gut microbiota of Han Chinese and ethnic minority groups from the same sites was more alike than that of the same ethnic minority groups from different sites, suggesting a stronger effect of geography than ethnicity on the whole microbial community^10^. In this study, the ethnicity-specific high abundance of *B. angulatum* and unclassified *Bifidobacterium* were not shared by all ethnic groups living in the same sites or geographical regions, suggesting that geography may have a weaker effect than ethnicity on some of the *Bifidobacterium* species. Nevertheless, both studies suggested that co-residence of different ethnic groups likely reduced ethnicity-specific characteristics. That is to say, such *Bifidobacterium* species might be more affected by factors uniquely related to certain ethnic groups but not all residents in the same region, for example, genetics, some ethnicity-specific food and cooking habits, etc. Regarding age, it is known that a stable gut microbiota resembling that of adults (microbiota maturation) occurs at age 3^38^. However, we found that the bifidobacterial community matured much later at middle adolescence, and age 30 was another important change point for some *Bifidobacterium* species. The importance of these two time points in the development of bifidobacterial community or microbiota has not been reported before, and further investigations is warranted to elucidate the related mechanisms.

The flourishing of bifidobacteria caused by wheat ingestion has long been acknowledged. As to the species level, such a phenomenon has been repeatedly observed for *B. adolescentis*, *B. longum*, and *B. angulatum* in short-term dietary intervention studies^18,19^. Our study revealed that at the population level, *B. adolescentis* and *B. longum* still behaved as wheat-responders, but not the other *Bifidobacterium* species. We focused on *B. adolescentis* to investigate the potential mechanism of growth advantage upon wheat ingestion, considering that the composition of *B. longum* subspecies was unclear in this study. Although there were multiple studies on the CAZymes of *Bifidobacterium* species^39,40^ and the relatively higher capacity of carbohydrate catabolism of *B. adolescentis*^29^, no systemic study on the CAZymes catabolizing wheat-specific polysaccharides of *B. adolescentis* has been reported. We screened 270 functional genes encoding glycosidases, and differences in arabinoxylan-degrading glycosidases between *B. adolescentis* and a non-responder *B. pseudocatenulatum* stood out. Particularly, genes encoding endo-1,4-beta-xylanase were only found in *B. adolescentis*. Therefore, the response of *B. adolescentis* to wheat may be partly attributed to its genomic advantages in utilizing arabinoxylan. This hypothesis needs to be tested through in vitro studies, including characterization of related enzymes and enzyme activities, as well as measurement of growth capacity of *Bifidobacterium* species upon culturing with arabinoxylan or other polysaccharides.

There are technical limitations in this study. The sequencing method using maker gene *groEL* has a low confidence in identifying *B. catenulatum*, and in differentiating subspecies belonging to *B. longum*, leading to the lack of profile of these *Bifidobacterium* spp.. In addition, this study could be improved by absolute quantification instead of using relative abundance in describing the bifidobacterial community. Nevertheless, this study revealed the population-level variation in the gut bifidobacterial community, identified several key affecting factors, and shed light on the mechanism of bifidobacterial community construction from the perspective of genome function. These findings highlight the importance of environmental and host factors in shaping the gut bifidobacterial community, and provide a fundamental basis for the precise screening, design, and application of probiotic bifidobacteria.

## Methods

### The cohort

A subset of a cohort including 2678 Chinese individuals in our previous study^10^, which had adequate fecal DNA material or bifidobacterial reads for downstream analysis, was included in this study, resulting in 1674 participants. Twenty-eight out of 34 provincial-level administrative divisions in China were represented, and one to five random sampling sites in each province were included. All participants had no self-reported gastrointestinal tract disorder or any other acute/chronic/recurrent medical conditions, and no use of antibiotics for at least 3 months prior to participation.

The study was approved by the Ethical Committee of Jiangnan University. Written informed consent was obtained from all participants or their legal representatives for minors.

### Amplicon sequencing and taxonomy classification

Genomic DNA extraction and amplicon library preparation targeting the *groEL* gene were performed as described before^23^. Sequencing was performed on the Illumina Miseq platform with the Miseq Reagent Kit V3 (CA, USA, PE300 mode). Reads were merged, de-multiplexed, quality-filtered, and further clustered into 8427 ZOTUs as described before^10^.

Taxonomy classification of ZOTU representative sequences was based on the National Center for Biotechnology Information (NCBI) Nucleotide Sequence Database (referred to as “NT”) downloaded on July 15, 2021, as well as an inhouse *Bifidobacterium groEL* gene database (referred to as “BIF”). To construct the BIF database, we downloaded all the *Bifidobacterium* spp. genomes that were available in the NCBI Reference Sequence Database (RefSeq) on August 20, 2021. The *groEL* gene was extracted, and then trimmed to the amplicon length (approximately 460 bp) using Cutadapt v2.11^41^ and the Bif-*groEL* primers. Duplicate sequences were removed using ElimDupes (https://www.hiv.lanl.gov/elimdupes.html), resulting in 309 unique sequences. Sequences of *Lactobacillus groEL* gene were obtained using the same method and used as an outgroup. Sequence identity in the BIF database was estimated after multiple alignments using MAFFT v7.487^42^, revealing that more than 99% of the sequence pairs in the BIF database had an identity higher than 75%, whereas the identity between *Bifidobacterium* and the outgroup was lower than 62% (Supplementary Fig. 1b).

ZOTU representative sequences were aligned to the NT and BIF databases using Megablast v2.9.0^43^ with the following settings: e value 1e-10, max_target_seqs 1000, qcov_hsp_perc 60, perc_identity 75. Taxonomy based on NT was obtained using the Least Common Ancestor (LCA) algorithm with MEGAN v6.12.2^44^ (--maxMatchesPerRead 2000, --minScore 100, --minSupport 1, --topPercent 10), and taxonomy based on BIF was obtained using the LCA algorithm with the top 10% BLAST hits. A total of 540 ZOTUs with no BLAST hits, and 6,437 ZOTUs having taxonomy (inferred from the NT database) not belonging to *Bifidobacterium* and maximum-bit-score-NT > maximum bit-score-BIF were discarded. Of the remaining 1450 ZOTUs, 866 of them were assigned the same taxonomy using either NT or BIF database, and for those assigned different taxonomies using NT and BIF, the taxonomy of each ZOTU was determined as follows: if maximum-bit-score-BIF = maximum-bit-score-NT, or if maximum-bit-score-BIF > maximum-bit-score-NT and maximum-bit-score-BIF*0.9 ≤ maximum-bit-score-NT, the one with higher taxonomy level between the two was used; if maximum-bit-score-BIF > maximum-bit-score-NT and maximum-bit-score-BIF*0.9 >maximum-bit-score-NT, taxonomy based on BIF was used; no ZOTUs had maximum-bit-score-BIF < maximum-bit-score-NT under this condition (Supplementary Table 2).

### Analysis of the bifidobacterial community

ZOTU representative sequences and sequences from the BIF database were aligned using MAFFT v7.487, and the phylogeny was inferred with IQ-TREE v2.1.4^45^ with 1000 bootstraps. The phylogenetic tree was visualized using iTOL v6^46^.

The ZOTU table was rarefied to 2,000 reads per sample for analyzing alpha and beta diversity. Observed ZOTUs, Faith’s PD, and Shannon index were calculated using QIIME2 v2018.10^47^. JSD was calculated using the phyloseq R package v1.32.0^48^. The non-rarefied ZOTU table was used for all the other analyses.

To estimate the worldwide gut bifidobacterial composition, we extracted data from the curatedMetagenomicData v3.4.1 R package^49^ by selecting healthy individuals, filtering out samples with redundant information, and filtering countries with less than 50 samples. A total of 29 *Bifidobacterium* species was obtained from 4516 samples collected in 14 countries (Supplementary Table 3). The latitude and longitude of sampling sites were obtained from LatLong (https://www.latlong.net/), and the latitude and longitude of the capitals of countries were used if sampling sites was not registered.

### Bifidobacterial strain isolation and genome analysis

Fecal samples were homogenized in sterile PBS and centrifuged at 2000 × *g* for 1 min, and the supernatant was diluted and spread on the DeMan-Rogosa-Sharpe agar plates supplemented with 0.05% (w/v) L-cysteine hydrochloride. The plates were then incubated at 37 °C in an anaerobic chamber with 10% (v/v) H_2_, 10% CO_2_ and 80% N_2_. The taxonomy of isolates was determined based on the 16 S rRNA gene as described before^34^.

Genomic DNA was extracted from each isolate using the Rapid Bacterial Genomic DNA Isolation Kit (Sangon Biotech Ltd., Shanghai, China). Libraries were prepared using the NEXTFLEX Rapid DNA-Seq Kit (PerkinElmer, TX, USA), and sequenced on the Illumina Hiseq X Ten platform (CA, USA, PE150 mode) to obtain no less than 100-fold genome coverage. Sequencing reads were subjected to adaptor trimming and quality filtering using fastp v0.20.0^50^. Clean reads were assembled using SOAPdenovo v2.04^51^, and inner gap filling and base correction were performed using GapCloser v1.12^52^. The distance between genomes was estimated using Mash v2.3^53^. To predict putative carbohydrate-active enzymes, genomes were aligned against the CAZy database^54^ using HMMER on the dbCAN2 meta server^55^ (threshold amino acid identity ≥30%, *E* value ≤ 1 × 10^−5^).

### Statistical analysis

To explore variables associated with the variation of *Bifidobacterium* community, nineteen variables having more than 500 samples with non-missing data were assessed using dbRDA separately with the *capscale* function of vegan R package v2.5-6^56^, and the adjusted *R*^2^ was extracted with the *RsquareAdj* function. Nine variables were further selected for inclusion in a stepwise dbRDA model using the *capscale* function. All eligible variables had to be significant in univariate dbRDA (except for sex, which was included regardless), and the variables had to have more than 800 samples with non-missing data. To characterize geography, we chose altitude, latitude, and longitude because they have better-generalized value than sampling site/province/geographical zone. For demography, we chose ethnicity, age, and sex. For urbanization status, we chose urbaun/rural/pastoral residence, because of its higher *R*^2^ than GDP and population density in univariate dbRDA. Staple food type belonging to the diet category and sampling month were also included. In the stepwise model, the adjusted R^2^ was calculated with the *ordiR2step* function using adjusted R^2^ as the stopping criterion (R2scope = TRUE).

To identify associations between abundances of *Bifidobacterium* species or alpha diversity indices and covariates, ridge regression, which is suitable for the scenario where the variables are highly correlated, was used with the *linearRidge* function of the ridge R package v3.3^57^. The above-mentioned nine representative variables were included in ridge models (Figs. 1e, 2a, 5a), except that staple food type was not included when identifying geographical zone-specific species (Fig. 2c), and this exclusion was due to the fact that <50% of the samples in II2, II4, III1, IV2 had information on staple food. Multiple linear regression with adjustment for age and sex was used to serve as a comparison with ridge regression wherever needed, and to identify associations for the World data. Reference groups for the categorical comparisons were female (sex), Han (ethnicity), rice (staple food type), rural (urban/rural/pastoral residence), and April (sampling month) in regression models.

Multiple tests were corrected using the Benjamini–Hochberg false discovery rate algorithm^58^ (*p*.adj value). *p* values smaller than 0.05 or *p*.adj values smaller than 0.1 were considered as statistically significant unless otherwise specified.

### Reporting summary

Further information on research design is available in the Nature Research Reporting Summary linked to this article.

### Supplementary information

Supplementary Figure

Supplementary Table

Reporting Summary

## Data Availability

The amplicon sequencing data reported in this study has been deposited in the Genome Sequence Archive in the National Genomics Data Center, Beijing Institute of Genomics (China National Center for Bioinformation), Chinese Academy of Sciences, under accession number PRJCA021377, is publicly accessible at https://bigd.big.ac.cn/gsa. The assembled bifidobacterial genomes are deposited in the NCBI GenBank database, with *B. adolescentis* genomes under accession no. PRJNA792507, PRJNA681061, PRJNA869357, and *B. psedudocatenulatum* genomes under accession no. PRJNA577207, PRJNA792507, PRJNA730686, and PRJNA681061.
