# Supplementary material for: Population-level variation in gut bifidobacterial composition and association with geography, age, ethnicity, and staple food
Source: NPJ Biofilms Microbiomes. 2023 Dec 12;9:98. doi: 10.1038/s41522-023-00467-4 (PMC10716157; doi:10.1038/s41522-023-00467-4)
Supplement: Supplementary file 1 — Supplementary Figure [file 41522_2023_467_MOESM1_ESM.pdf]

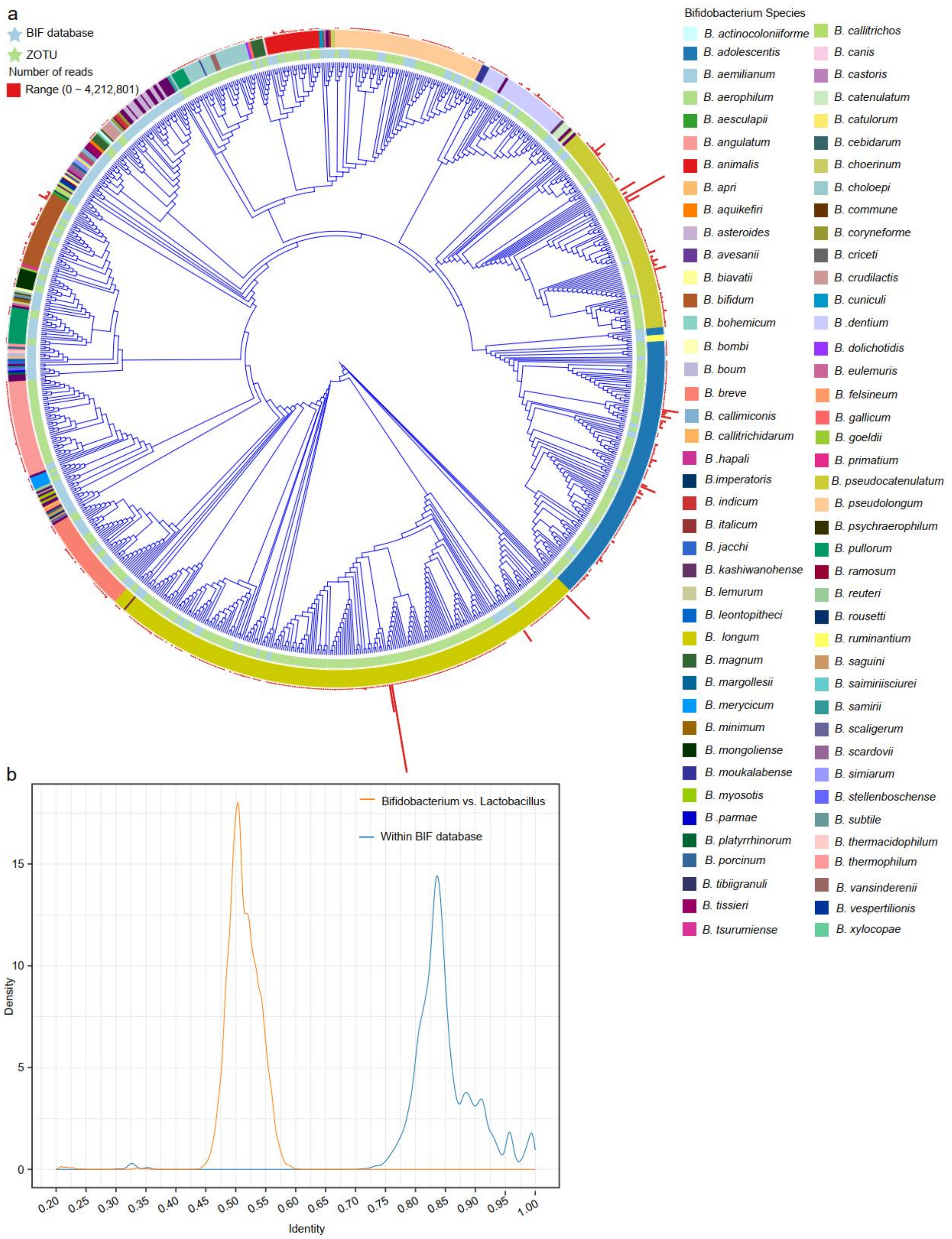

Supplementary Figure 1. Diversity of the bifidobacterial *groEL* gene.

(a) The phylogenetic tree of *groEL* gene of *Bifidobacterium* species. A total of 309 unique sequences of 82 *Bifidobacterium* species from the BIF database (indicated with light blue in the inner circle), as well as 604 ZOTU representative sequences of 17 species found in the Chinese gut (light green) were included. The number of reads mapped to each ZOTU is shown in the outer circle. (b) Density curve showing the sequence identity of *groEL* gene within the BIF database (blue), as well as between the BIF database and the outgroup *Lactobacillus* (orange).

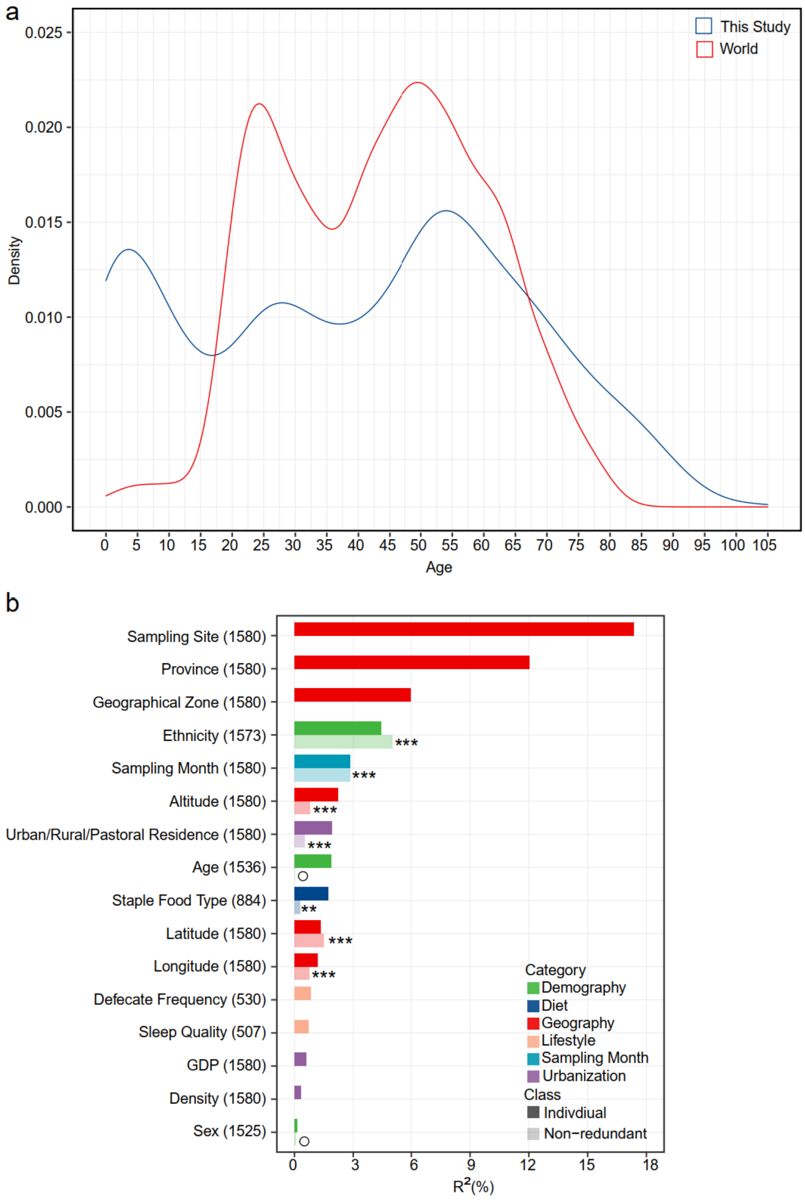

Supplementary Figure 2. Age distribution in this cohort and the World data, as well as covariates associated with the beta diversity of the gut bifidobacterial community.

(a) Density curve showing the age distribution in this cohort (blue) and the World data (red). (b) Covariates correlated with the beta diversity of the gut bifidobacterial community estimated with JSD. Nineteen variables with more than 500 samples having corresponding data were analyzed using distance-based redundancy analysis (dbRDA) separately, and variance explained by each of these variables is indicated with darker colors; 17 covariates with p.adj < 0.1 are shown. Nine representative variables with more than 800 samples having corresponding data were analyzed using a stepwise dbRDA, and the non-redundant variance explained by each of these variables is indicated with lighter colors; *p.adj < 0.1, ***p.adj < 0.01, and insignificant covariates are indicated with an open circle; n = 884. The number of samples is indicated in brackets following the name of each covariate.

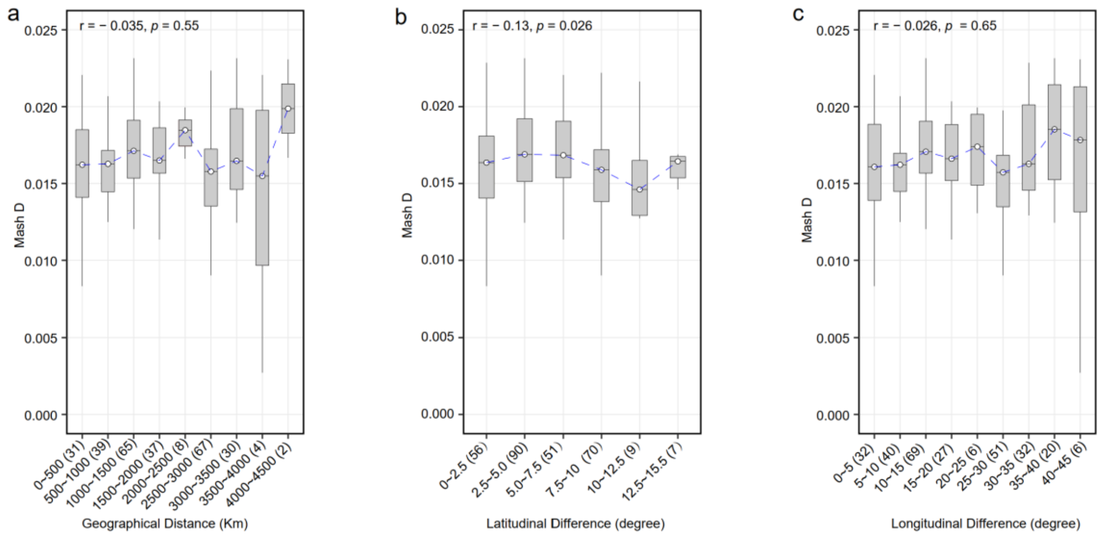

Supplementary Figure 3. Mash distance between *B. adolescentis* genomes in different geographical groups.

(a-c) Mash distance between *B. adolescentis* genomes in different geographical distance (a), latitudinal difference (b), and longitudinal difference (c) groups. The center line of the boxplot represents the median, box limits represent upper and lower quartiles, and whiskers represent 1.5x interquartile range. The associations between geographical factors and Mash distance were evaluated using Pearson's correlation test, and the coefficient and *p* values are shown.

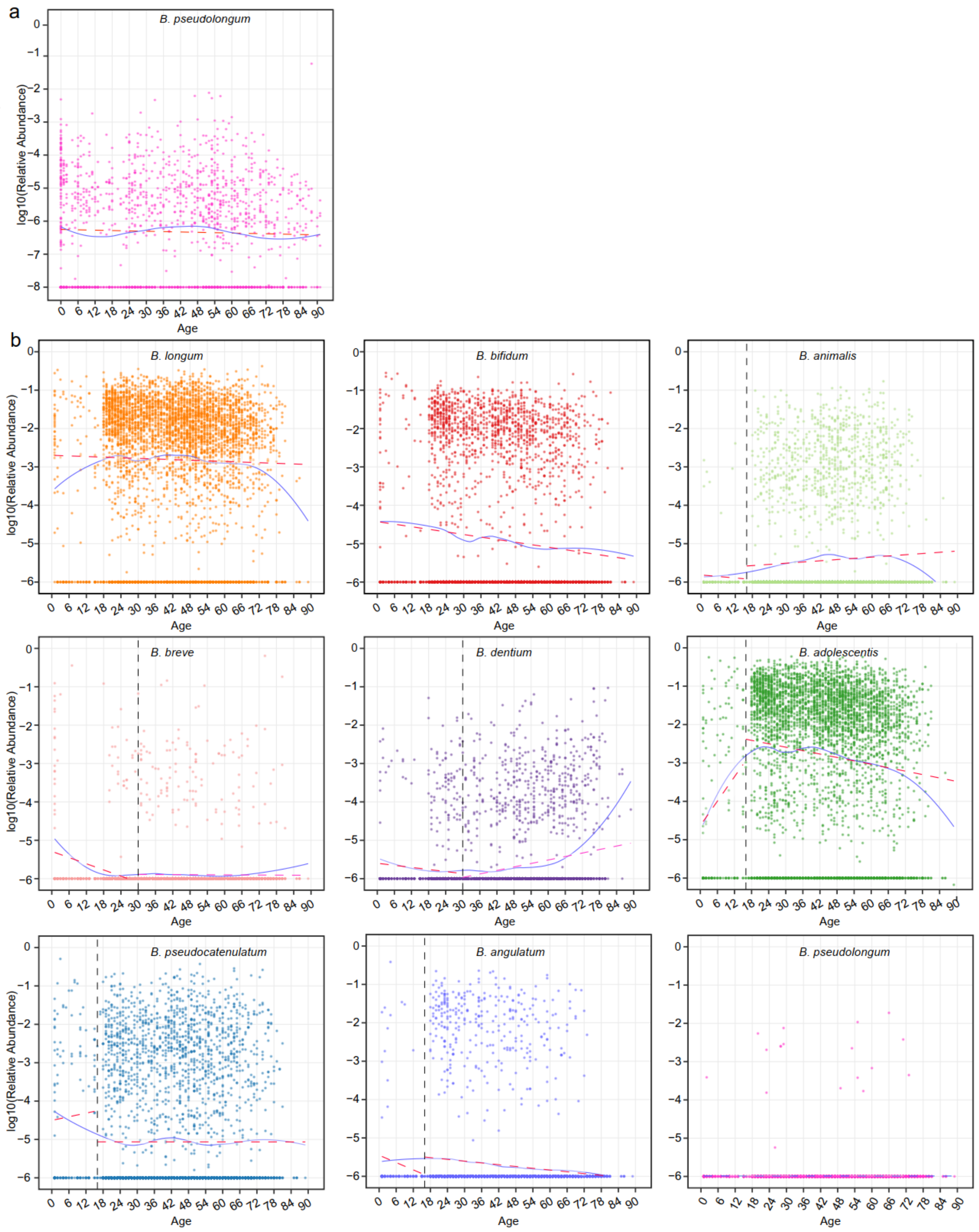

Supplementary Figure 4. Changes of the gut *Bifidobacterium* species along aging in this study and the World data.

(a) Relative abundance of *B. pseudolongum* in this study. n = 1,536. (b) Relative abundances of *Bifidobacterium* species in the World data. Each dot represents a sample. Fitting curves of loess regression models are indicated with blue lines, and those of linear regression models are indicated with red dotted lines. Black vertical dotted lines indicate key changing points (age 16 or 30) in the development of bifidobacterial community. n = 4,516.

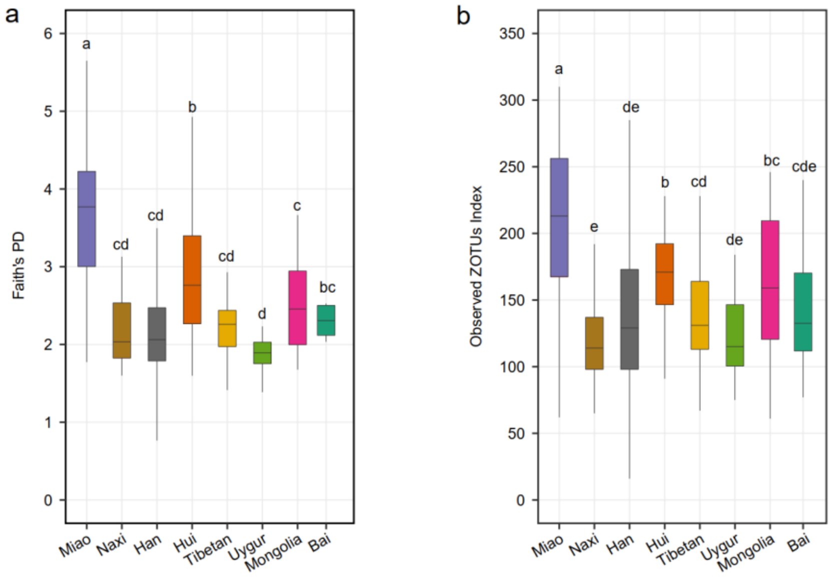

Supplementary Figure 5. Alpha diversity of the bifidobacterial community in different ethnic groups.

(a-b) Faith's PD (a) and Observed ZOTUs (b) in different ethnic groups. Different letters on the top indicate significant differences (p.adj < 0.1, one-way ANOVA and Fisher's Least Significant Difference test). In a-b, Han, n =1,349; Tibetan, n = 93; Bai, n = 14; Miao, n = 36; Naxi, n = 33; Uygur, n = 31; Hui, n = 72; Mongolian, n = 30.

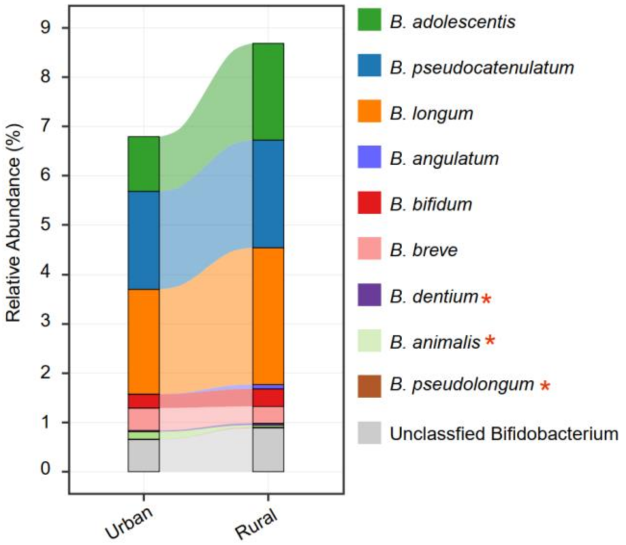

Supplementary Figure 6. Differences in the composition of gut bifidobacterial community between urban and rural populations. Mean values are shown. *p.adj < 0.1, ridge regression with adjustment for altitude, latitude, longitude, age, sex, ethnicity, sampling month, and staple food type. Urban, n = 237; Rural, n = 629.
