# Supplementary material for: Population-level variation in gut bifidobacterial composition and association with geography, age, ethnicity, and staple food
Source: NPJ Biofilms Microbiomes. 2023 Dec 12;9:98. doi: 10.1038/s41522-023-00467-4 (PMC10716157; doi:10.1038/s41522-023-00467-4)
Supplement: Supplementary file 3 — Reporting Summary [file 41522_2023_467_MOESM3_ESM.pdf]

nature portfolio

Corresponding author(s): Mingkun Li

Last updated by author(s): Jul 15, 2023

Reporting Summary

Nature Portfolio wishes to improve the reproducibility of the work that we publish. This form provides structure for consistency and transparency in reporting. For further information on Nature Portfolio policies, see our Editorial Policies and the Editorial Policy Checklist.

Statistics

For all statistical analyses, confirm that the following items are present in the figure legend, table legend, main text, or Methods section.

n/a	Confirmed	
☐	☒	The exact sample size (*n*) for each experimental group/condition, given as a discrete number and unit of measurement
☐	☒	A statement on whether measurements were taken from distinct samples or whether the same sample was measured repeatedly
☐	☒	The statistical test(s) used AND whether they are one- or two-sided<br>*Only common tests should be described solely by name; describe more complex techniques in the Methods section.*
☐	☒	A description of all covariates tested
☐	☒	A description of any assumptions or corrections, such as tests of normality and adjustment for multiple comparisons
☐	☒	A full description of the statistical parameters including central tendency (e.g. means) or other basic estimates (e.g. regression coefficient) AND variation (e.g. standard deviation) or associated estimates of uncertainty (e.g. confidence intervals)
☐	☒	For null hypothesis testing, the test statistic (e.g. *F*, *t*, *r*) with confidence intervals, effect sizes, degrees of freedom and *P* value noted<br>*Give P values as exact values whenever suitable.*
☒	☐	For Bayesian analysis, information on the choice of priors and Markov chain Monte Carlo settings
☒	☐	For hierarchical and complex designs, identification of the appropriate level for tests and full reporting of outcomes
☐	☒	Estimates of effect sizes (e.g. Cohen's *d*, Pearson's *r*), indicating how they were calculated

Our web collection on statistics for biologists contains articles on many of the points above.

Software and code

Policy information about availability of computer code

Data collection	The gut bifidobacterial composition of 4516 worldwide samples was extracted from the curatedMetagenomicData v3.4.1 R package; the latitude and longitude of corresponding sampling sites were obtained from LatLong (https://www.latlong.net/).
Data analysis	The groEL gene were extracted and trimmed using Cutadapt v2.11;<br>Duplicate sequences were removed using ElimDupes;<br>Multiple alignment of sequences was performed with MAFFT v7.487;<br>ZOTU representative sequences were aligned to databases using Megablast v2.9.0;<br>Taxonomy of ZOTUs was obtained using the Least Common Ancestor algorithm with MEGAN v6.12.2;<br>Phylogeny was inferred with IQ-TREE v2.1.4;<br>Phylogenetic tree was visualized using iTOL v6;<br>Observed ZOTUs, Faith's PD, and Shannon index were calculated using QIIME2 v2018.10;<br>JSD was calculated using the phyloseq R package v1.32.0;<br>envfit and PERMANOVA analyses were performed using the vegan R package v2.5-6;<br>Sequencing reads of bifidobacterial isolates were subjected to adaptor trimming and quality filtering using fastp v0.20.0;<br>Clean reads were assembled using SOAPdenovo v2.04;<br>Inner gap filling and base correction were performed using GapCloser v1.12;<br>Distance between genomes was estimated using Mash v2.3;<br>Genomes were aligned against the CAZy database using HMMER on the dbCAN2 meta server.

For manuscripts utilizing custom algorithms or software that are central to the research but not yet described in published literature, software must be made available to editors and reviewers. We strongly encourage code deposition in a community repository (e.g. GitHub). See the Nature Portfolio guidelines for submitting code & software for further information.

Data

Policy information about availability of data

All manuscripts must include a data availability statement. This statement should provide the following information, where applicable:

- Accession codes, unique identifiers, or web links for publicly available datasets
- A description of any restrictions on data availability
- For clinical datasets or third party data, please ensure that the statement adheres to our policy

The amplicon sequencing data reported in this study has been deposited in the Genome Sequence Archive in National Genomics Data Center, Beijing Institute of Genomics (China National Center for Bioinformation), Chinese Academy of Sciences, under accession number HRA001812 that are publicly accessible at https://bigd.big.ac.cn/gsa. The assembled bifidobacterial genomes are deposited in the NCBI GenBank database, with B. adolescentis genomes under accession no. PRJNA792507, PRJNA681061, PRJNA869357, and B. psedudocatenulatum genomes under accession no. PRJNA577207, PRJNA792507, PRJNA730686, and PRJNA681061.

Research involving human participants, their data, or biological material

Policy information about studies with human participants or human data. See also policy information about sex, gender (identity/presentation), and sexual orientation and race, ethnicity and racism.

Reporting on sex and gender	This study included 712 males and 813 females. The sex information was confirmed through participants' Resident Identity cards.
Reporting on race, ethnicity, or other socially relevant groupings	The cohort comprised 1,349 Han Chinese, 309 individuals from seven ethnic minority groups (Tibetan, 93; Hui, 72; Miao, 36; Naxi, 33; Uygur, 31; Mongolian, 30; Bai, 14) and 16 individuals without ethnic information. The ethnic information was confirmed through participants' Resident Identity cards.<br>Age, sex, and ethnicity were included as confounding factors in linear regression and ridge regression analyses; the non-redundant effect of different covariates was evaluated using dbRDA.
Population characteristics	The study included 1,674 volunteers without apparent diseases (referred to as "healthy"). The age range was 0.01-103 years with a median of 41.
Recruitment	The 60 sampling sites from 28 provinces were chosen to represent different geographic conditions and urbanization status in China. Participants were randomly recruited from each sampling site.
Ethics oversight	The study was approved by the Ethical Committee of Jiangnan University. Written informed consents were obtained from all participants or their legal representatives for minors.

Note that full information on the approval of the study protocol must also be provided in the manuscript.

Field-specific reporting

Please select the one below that is the best fit for your research. If you are not sure, read the appropriate sections before making your selection.

☒ Life sciences ☐ Behavioural & social sciences ☐ Ecological, evolutionary & environmental sciences

For a reference copy of the document with all sections, see nature.com/documents/nr-reporting-summary-flat.pdf

Life sciences study design

All studies must disclose on these points even when the disclosure is negative.

Sample size	This study included 1674 human subjects (one fecal sample from each subject was used), making it the largest cohort study to date investigating the configurations of gut bifidobacterial across a broad range of geography, climate, topography, age, dietary habits, and culture. Since there was rare research on the population-level variation in the gut bifidobacterial community, it was hard to predetermine sample size for this research purpose.
Data exclusions	Samples were chosen from a cohort including 2,678 Chinese individuals in our previous study, and a sample was excluded if 1) no adequate fecal DNA material was available, 2) the number bifidobacterial reads from sequencing was less than 2000, 3) the participant had gastrointestinal tract disorder or any other acute/chronic/recurrent medical conditions, 4) the participant had taken antibiotics in the three months prior to paricipation.
Replication	We collected bifidobacterial profiles in the existing metagenomic data worldwide (4516 healthy individuals from 14 countries), and replicated our analyses wherever applicable. Important findings from this study, including the association between Bifidobacterium species and geography and age, were replicable in the worldwide dataset.
Randomization	Samples were allocated into groups based on their demographic features or associated environmental factors during data analysis, which did not require randomization.

Blinding	This study does not involve any treatment groups that would require blinding during assignment of subjects to groups.

Reporting for specific materials, systems and methods

We require information from authors about some types of materials, experimental systems and methods used in many studies. Here, indicate whether each material, system or method listed is relevant to your study. If you are not sure if a list item applies to your research, read the appropriate section before selecting a response.

Materials & experimental systems

n/a	Involved in the study
☒	☐ Antibodies
☒	☐ Eukaryotic cell lines
☒	☐ Palaeontology and archaeology
☐	☒ Animals and other organisms
☒	☐ Clinical data
☒	☐ Dual use research of concern
☒	☐ Plants

Methods

n/a	Involved in the study
☒	☐ ChIP-seq
☒	☐ Flow cytometry
☒	☐ MRI-based neuroimaging

Animals and other research organisms

Policy information about studies involving animals; ARRIVE guidelines recommended for reporting animal research, and Sex and Gender in Research

Laboratory animals	The study did not involve laboratory animals.
Wild animals	The study did not involve wild animals.
Reporting on sex	The study used bifidobacterial isolates and thus sex is irrelevant.
Field-collected samples	Bifidobacterial strains were isolated from human fecal samples, incubated at 37 ℃ in an anaerobic chamber with 10% (v/v) H2, 10% CO2 and 80% N2.
Ethics oversight	The study used bifidobacterial isolates and thus ethics is irrelevant.

Note that full information on the approval of the study protocol must also be provided in the manuscript.
